# Recent Approaches for Wound Treatment

**DOI:** 10.3390/ijms24065959

**Published:** 2023-03-22

**Authors:** Cinzia Pagano, César Antonio Viseras Iborra, Luana Perioli

**Affiliations:** 1 Department of Pharmaceutical Sciences, University of Perugia, 06123 Perugia, Italy; cinzia.pagano@unipg.it; 2 Department of Pharmacy and Pharmaceutical Technology, Faculty of Pharmacy, University of Granada, 18071 Granada, Spain; cviseras@ugr.es

Wounds are a serious global health problem. In particular, chronic wounds are the most challenging because their management remains difficult and the available treatments are often ineffective. This is responsible for a serious impact on patient quality of life as well as on healthcare systems, as in many cases, patient hospitalization is required, resulting in high costs. The total Medicare spending estimates for all wound types ranges from USD 28.1 to USD 96.8 billion [1].

The aim of this Special Issue is to present new approaches as valuable alternatives that could be useful in the treatment and management of wounds. The proposals take into account efficacy and safety and are environmentally friendly.

The strategies are varied and the treatments can be performed using many different approaches. The proposed strategies include new ingredients (e.g., A.P.I. and polymers) able to stimulate wound healing, new delivery systems and new formulations, as well as a new understanding of the stimulation of physiological biochemical pathways. New state-of-the-art technologies as well as diagnostic tools are also presented.

Effective therapies are decided after appropriate diagnoses and an assessment of the specific problems for each patient.

Curti et al. [2] suggested an automated model able to perform a deep analysis of wounds from images acquired from smartphones using an app developed *ad hoc*. This method is based on an active semi-supervised learning training of a convolutional neural network. It was proven to be a suitable instrument for clinical practice and a valuable support for clinicians in choosing the most appropriate treatment.

Sim et al. [3] evaluated the effect of pH value variation in the promotion of wound healing. In particular, they observed that the incubation of human immortalized keratinocytes (HaCaT) and human foreskin fibroblasts (HFF) with acidic buffer promotes cell proliferation and migration, fundamental processes for wound healing.

Bianchi et al. [4] purposed nano-fibrous scaffolds having anti-inflammatory, antibacterial and antioxidant activities. The scaffolds are characterized by aligned nanofibers able to mimic the tendon structure and to promote reconstruction and healing. The nanofibers were produced by electrospinning, an innovative technique, using a biodegradable and biocompatible synthetic polymer, poly(butyl cyanoacrylate) (PBCA), combined with copper oxide nanoparticles and caseinphosphopeptides (CPP).

With the aim of finding useful tools from natural sources, Gębarowski et al. [5] investigated the benefits of wound dressings made from flax fibers and observed that they are efficacious in the promotion of tissue regeneration.

Kua et al. [6] studied the potential use of human umbilical cord lining epithelial cells for treating cutaneous wounds. In vivo studies assessed their ability to promote wound healing.

Smart technologies could be useful instruments for customized treatments of wounds.

Ceccarini et al. [7] purposed functionalized silk fibers covalently linked to an arginine–glycine–aspartic acid (RGD) peptide to 3D print grid-like piezoresistors with wound healing and sensing properties. The peptide-modified silk fibroin exhibited wound healing capacity and piezoresistive properties, and additionally demonstrated a sensitivity to humidity.

Often, infections are one of the most common complications of chronic wounds. Consequently, the healing process is further delayed, with serious consequences for the patient. The resolution of infections is thus an important approach in the promotion of wound healing. However, the use of conventional antibiotics is often unsuccessful due to antibiotic resistance. In 2019, 1.27 million deaths were caused by treatment-resistant infections [8]. Thus, the search for new and effective alternatives is necessary.

Lin et al. [9], for example, developed metal–organic frameworks (MOFs), which are systems consisting of metal ions (copper in this case) coordinated to organic ligands to form one-, two-, or three-dimensional structures. As a result of their peroxidase (POD)-like activity, hydroxyl radicals are produced from hydrogen peroxide. These radicals, together with Cu, possess antibacterial activity, responsible for the fast resolution of infections and thus the acceleration of wound healing.

Bąchor et al. [10] developed new isoxazole derivatives which showed antibiofilm activity toward *Staphylococcus aureus*, representing a valuable alternative to conventional broad-spectrum antibiotics currently used in therapy which often suffer from antimicrobial resistance.

Di Lodovico et al. [11] developed graphene oxide compounds activated by light-emitting diodes, which demonstrated antimicrobial activity against *Staphylococcus aureus*- and *Pseudomonas aeruginosa*-resistant strains in a dual-species biofilm.

The necessity to evaluate the efficacy of most treatment options also poses a problem in that in vivo studies are limited and, in the case of wounds, injuries to the skin must be induced in animals. Cialdai et al. [12] purposed an ex vivo model for wound healing studies based on a human skin specimen (skin biopsies) mounted in a special chamber equipped with a device able to monitor tissues changes, avoiding the unnecessary use of animals.
